# Renal tubulointerstitial lesions: a prognostic marker in idiopathic membranous nephropathy

**DOI:** 10.1080/0886022X.2025.2501379

**Published:** 2025-05-22

**Authors:** Yonghua Liu, Xiuhua Ma, Min Yu, Xiaoling Zhou

**Affiliations:** a Department of Nephrology, General Hospital of Ningxia Medical University, Yinchuan, China; b Department of Nephrology, School of Clinical Medicine, Ningxia Medical University, Yinchuan, China

**Keywords:** Idiopathic membranous nephropathy, renal tubulointerstitial lesions, clinicopathological, prognosis

## Abstract

**Objective:**

To evaluate the impact of renal tubulointerstitial lesions (TIL) on clinical and pathologic outcomes and prognosis in patients with idiopathic membranous nephropathy.

**Methods:**

A retrospective cohort study was performed on 582 patients with idiopathic membranous nephropathy. Patients were divided into two groups based on the presence or absence of TIL as determined by renal biopsy: TIL– (258 cases) and TIL+ (324 cases). Kaplan-Meier survival curves and Cox regression models were used to analyze the influence of TIL on renal prognosis. Logistic regression models were used to further identify risk factors associated with the development of TIL.

**Results:**

Patients in the TIL+ group were predominantly male, older, and had a higher prevalence of hypertension, hyperlipidemia, nephrotic syndrome, microscopic hematuria, and immunosuppressive therapy compared to the TIL– group. In addition, triglycerides, blood urea nitrogen, and 24-hour urine protein were significantly higher in the TIL+ group, while albumin and estimated glomerular filtration rate were lower (*p* < 0.05). Pathologic staging revealed more severe glomerulosclerosis lesions and renal artery intimal thickening in the TIL+ group. After a median follow-up of 45 months, IMN patients in the TIL+ group had a lower disease remission rate and worse renal prognosis as demonstrated by Kaplan-Meier survival curves and Cox regression modeling. Logistic regression modeling identified hypertension, globular/segmental glomerulosclerosis, and intimal thickening of small renal arteries as independent risk factors for TIL in patients with idiopathic membranous nephropathy.

**Conclusion:**

TIL is frequently associated with idiopathic membranous nephropathy, with more severe clinical manifestations and pathologic features, and idiopathic membranous nephropathy patients with TIL have a lower disease remission rate and worse overall renal prognosis. Hypertension, globular/segmental glomerulosclerosis, and intimal thickening of small renal arteries are independent risk factors for the development of TIL in patients with idiopathic membranous nephropathy.

## Introduction

Membranous nephropathy is a common primary glomerular disease, especially in adults with nephrotic syndrome. Especially in China, membranous nephropathy has emerged as the primary glomerular disease with the fastest growing incidence, emphasizing the urgency of understanding its pathogenesis and prognosis [1]. Membranous nephropathy is broadly classified into idiopathic membranous nephropathy (IMN) and secondary membranous nephropathy, with the distinction based on the clarity of the underlying etiology [2]. IMN, an immune-mediated disease, is characterized by the deposition of immune complexes beneath the epithelial cells of the glomerular basement membrane, resulting in diffuse thickening of the basement membrane [3]. Clinical outcomes for IMN patients are highly variable, with approximately one-third achieving spontaneous remission, one-third having persistent proteinuria, and the remaining one-third progressing to end-stage renal disease [4].

Previous studies have identified factors such as age, blood creatinine levels, proteinuria and anti-PLA2R antibodies as significant predictors of IMN prognosis [5–7]. Recent evidence suggests that IMN patients with tubulointerstitial lesions (TIL) have more severe clinical manifestations and poorer prognosis, highlighting the potential of TIL as a critical predictor of persistent renal decline in IMN [8,9]. In the context of primary glomerulonephritis, the relationship between tubular disease and the prognosis of chronic kidney disease is more pronounced than that of glomerulonephritis [10], but the role of renal pathological damage, particularly TIL, in predicting disease progression has been less explored.

This study aims to retrospectively analyze the clinical and pathological data of IMN patients diagnosed by renal biopsy in our hospital. We will compare the clinical and pathologic characteristics of IMN patients with and without TIL and explore the impact of TIL on the prognosis of IMN patients. Our goal is to increase the clinical awareness of TIL in IMN patients, thereby improving the quality of care and improving the prognosis and quality of life of patients with MN, ultimately reducing the burden on healthcare systems.

## Methods

### Study population

**Figure 1.**
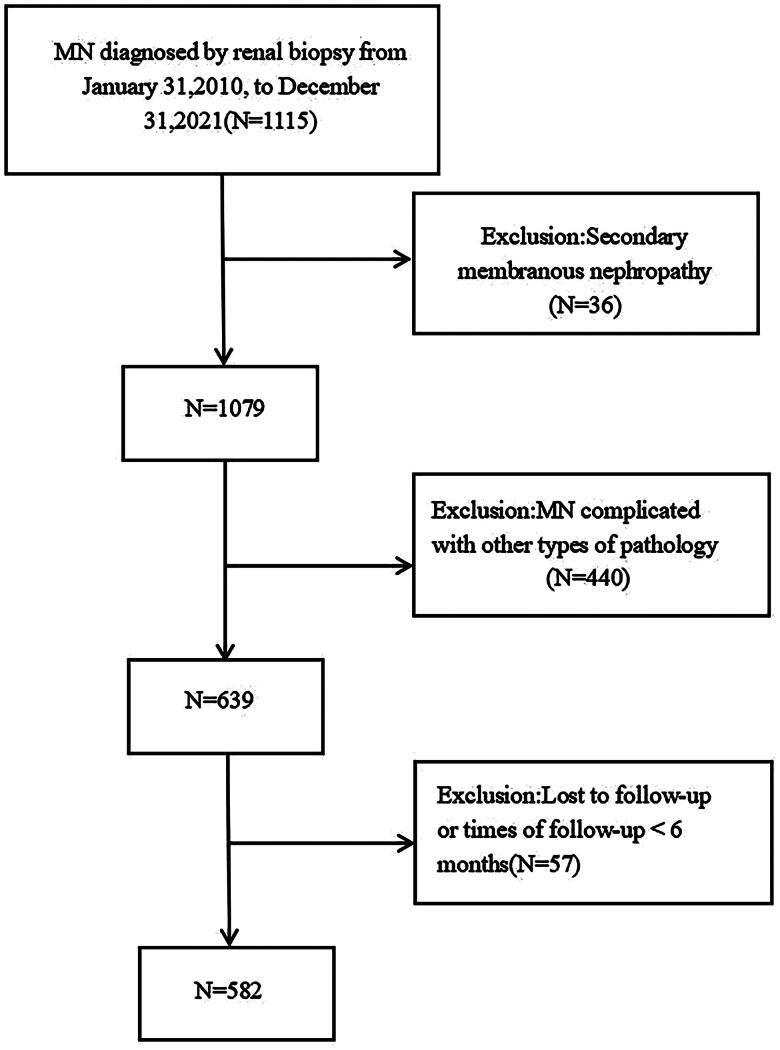
Eligibility and exclusion criteria for the study population.

This retrospective study included a total of 582 adult patients diagnosed with idiopathic membranous nephropathy by renal biopsy at the Department of Nephrology, General Hospital, Ningxia Medical University, between January 2010 and December 2021. Inclusion criteria: adult patients with IMN confirmed by renal biopsy. Patients who were followed up for at least 6 months through hospitalization, outpatient visits, and telephone consultations.Exclusion criteria: patients with a history of infectious diseases (e.g. patients with a history of infectious diseases (e.g. hepatitis B, hepatitis C), autoimmune diseases, drug-induced conditions, or tumors that could lead to secondary membranous nephropathy, as determined by history, laboratory tests, and imaging studies. Patients with IMN combined with other pathologic types, such as diabetic nephropathy or IgA nephropathy. Patients with incomplete clinical and follow-up data. The entire screening process is shown in Figure 1.

### Clinical and laboratory parameters

Clinical and laboratory parameters, including sex, age, blood pressure, complications (hypertension, diabetes, hyperuricemia, hyperlipidemia), treatment, microscopic hematuria, hemoglobin, 24-h urine protein, blood urea nitrogen, and serum creatinine, uric acid, albumin, triglycerides, total cholesterol and high-density lipoprotein cholesterol, low-density lipoprotein cholesterol, estimated glomerular filtration rate (eGFR) as estimated by the Chronic Kidney Disease Epidemiology Collaboration equation.

### Evaluation of histopathology

Kidney biopsies from all 582 IMN patients were evaluated using a standardized protocol that included light microscopy, immunofluorescence and electron microscopy. Each biopsy included at least 10 glomeruli for comprehensive evaluation. Ehrenreich-Churg staging was used for the pathologic diagnosis of IMN. Two renal pathologists independently scored the biopsies using the Katafuchi scoring system [11], which evaluates renal interstitial fibrosis, inflammation, and tubular atrophy on a scale of 0–3 (0 = none, 1 = mild, 2 = moderate, 3 = severe). The total Tubulointerstitial Lesion score ranged from 0 to 9, with scores categorized as mild (1–3), moderate (4–6), and severe (7–9). In our study, TIL were predominantly mild (315/324), with very few moderate cases (9/324) and no severe cases. Among these, interstitial fibrosis accounted for 26.2% (85/324), interstitial inflammatory cell infiltration for 79.0% (256/324), and tubular atrophy for 79.0% (256/324). Given the predominance of mild TIL cases in our study, patients were divided into TIL– (no TIL) and TIL+ (presence of TIL) groups for further analysis.

### Treatment and follow-up records

Treatment principles and remission criteria were based on the 2024 Kidney Disease Improving Global Prognosis Organization (KDIGO) guidelines [12]. Complete remission (CR): 24-h urine protein <0.3 g, ALB ≥35 g/L and normal serum creatinine; partial remission (PR): 24-h urine protein 0.3–3.5 g with >50% decrease from baseline, normal or improved albumin, and stable serum creatinine; no remission (NR): less than 50% decrease in urine protein volume or decline in renal function.

### Endpoints

The primary endpoint of the study was all-cause mortality. The secondary endpoint was a composite renal endpoint defined as either *a* > 30% decrease in estimated glomerular filtration rate from baseline or initiation of end-stage renal disease treatment.

#### Follow-up and definitions

Follow-up was completed through June 1, 2021, or the occurrence of an endpoint event.

Kidney survival was measured from the date of renal biopsy to the study endpoint or the last available follow-up data if no endpoint was reached.

### Statistical analyses

Data were analyzed using SPSS version 26.0 (IBM Corp., Chicago, IL, USA). Continuous variables with normal distribution were presented as mean ± standard deviation (SD) and compared using t-tests. Non-normally distributed continuous variables were expressed as median (interquartile range, IQR) and compared using the rank sum test. Count data were expressed as numbers (percentages) and compared using the chi-squared test or Fisher’s exact test, as appropriate.

Kaplan-Meier survival curves were constructed to estimate survival probabilities, and the log-rank test was used to compare differences between groups. The Cox proportional hazards model was used to assess the effect of renal TIL on renal prognosis. Logistic regression analysis was performed to identify risk factors for the development of TIL.

A two-tailed *p* value of < 0.05 was considered statistically significant.

## Results

### Clinical data

A total of 582 patients diagnosed with idiopathic membranous nephropathy were enrolled in this study and divided into two groups based on the presence of tubulointerstitial lesions: 258 (44.3%) in the TIL– group and 324 (55.7%) in the TIL+ group. Demographic and clinical characteristics differed significantly between the groups, with the TIL+ group having a higher proportion of male patients, older age, and elevated blood pressure (*p* < 0.05). In addition, the incidence of hypertension and hyperlipidemia was significantly higher in the TIL+ group (*p* < 0.05). Our study indicated that a higher proportion of patients in the TIL–group receiving renin-angiotensin-aldosterone system inhibitors (RASSI), whereas a higher ­proportion of patients in the TIL+ group were receiving immunosuppressive therapy (*p* < 0.05), as shown in Supplementary Material Table S1. The TIL+ group also had a higher prevalence of microscopic hematuria, nephrotic syndrome, and elevated levels of triglycerides, blood urea nitrogen, and 24-h urine protein quantification, as well as a significantly lower estimated glomerular filtration rate (*p* < 0.01). These findings are detailed in Table 1.

**Table 1.** Comparison of baseline clinical data of IMN patients between the two groups.

Variable	TIL– group (*n* = 258)	TIL+ group (*n* = 324)	*p*
Male (number, percentage)	147 (57.0%)	212 (65.4%)	0.04
Age (years)	46.19 ± 12.51	50.58 ± 11.92	<0.001
Systolic Blood Pressure (mmHg)	129.22 ± 17.05	133.1 ± 20.00	0.02
Diastolic Blood Pressure (mmHg)	84.98 ± 11.35	87.25 ± 13.07	0.03
Mean Arterial Pressure (mmHg)	99.73 ± 12.31	102.52 ± 14.07	0.01
Complication (n, %)			
Hypertension	91 (35.3%)	174 (53.7%)	<0.001
Diabetes	20 (7.8%)	33 (10.2%)	0.31
Hyperuricemia	60 (23.3%)	7 9(24.4%)	0.75
Hyperlipidemia	228 (88.4%)	303 (93.5%)	0.03
Microscopic Hematuria	159 (55.8%)	231 (65.3%)	0.01
Hemoglobin (g/L)	139.25 ± 20.52	139.49 ± 18.58	0.88
Nephrotic Syndrome (n, %)	106 (37.2%)	161 (45.5%)	0.04
Albumin (g/L)	26.04 ± 6.69	24.19 ± 6.88	0.001
Urinary protein excretion (g/24h)	3.17 (2.04,4.50)	3.62 (2.49,5.26)	0.002
Triglycerides (mmol/L)	2.36 (1.49,3.56)	2.59 (1.81,3.88)	0.01
Total Cholesterol (mmol/L)	6.70 (5.31,8.35)	6.77 (5.72,8.05)	0.42
High-Density Lipoprotein Cholesterol (mmol/L)	1.28 (1.04,1.64)	1.23 (1.01,1.50)	0.10
Low-Density Lipoprotein Cholesterol (mmol/L)	3.81 (2.72,4.85)	4.02 (3.02,4.89)	0.13
Uric Acid (μmol/L)	339.50 (275.00,415.25)	352.00(295.00,418.50)	0.21
Blood Urea Nitrogen (mmol/L)	4.62 (3.76,5.61)	5.06 (4.05,6.25)	0.001
eGFR (mL/min/1.73 m^2^)	126.08 (105.73,148.16)	112.87 (91.11,136.30)	<0.001

eGFR, estimated glomerular filtration rate. *Between the TIL– group and the TIL+ group.

### Histopathological data

This study evaluated the histopathologic characteristics of patients with idiopathic membranous nephropathy and showed a predominance of stage I and stage II in both the TIL– and TIL+ groups. However, a significant difference in the distribution of pathologic stages was observed, with a lower proportion of stage I patients and a higher proportion of stage III-IV patients in the TIL+ group (*p* < 0.01). In addition, the TIL+ group had a higher prevalence of glomerulosclerosis/segmental glomerulosclerosis and intimal thickening of small renal arteries compared to the TIL– group (*p* < 0.01). Notably, 97.2% of patients in this study presented with mild TIL, with no cases of severe TIL, indicating that while TIL is a common finding in IMN, the severity of these lesions is generally mild, as shown in Supplementary Material Table S2.

### Treatment and outcome

In our study of 582 patients with idiopathic membranous nephropathy, 430 patients underwent regular follow-up and maintained treatment for 6 months, with 190 (44.2%) in the TIL– group and 240 (55.8%) in the TIL+ group. After the 6-month treatment regimen, significant reductions in 24-h urine protein and albumin levels were observed in both groups compared to baseline. However, the overall remission rate, defined as the combination of complete and partial remission, was significantly lower in the TIL+ group compared to the TIL– group, with statistical significance (*p* < 0.05). This indicates that after an initial 6-month treatment period, IMN patients in the TIL+ group had a poorer treatment response and were less likely to achieve clinical remission. These results are detailed in Supplementary Material Table S3.

### Prognostic analysis

Membranous nephropathy, a total of 151 (25.9%) patients experienced end-point events. All-cause mortality occurred in 12 cases, cardiovascular events in 6, serious infections in 3, cancer in 2 and a single case due to a car accident. Renal composite endpoints were observed in 139 cases, primarily characterized by a greater than 30% decline in estimated glomerular filtration rate from baseline in 135 cases and end-stage renal disease in 4 cases, of which 1 required peritoneal dialysis and 3 underwent hemodialysis; no renal transplants were reported. The incidence of endpoint events was significantly higher in the TIL+ group compared to the TIL– group (*p* < 0.05). These results indicate that the TIL+ group had a worse renal prognosis and a significantly increased risk of endpoint events, while all-cause mortality was not significantly different between the two groups. A detailed comparison of endpoint events is shown in Table 2.

**Table 2.** Comparison of endpoint events between two groups of IMN patients during follow-up.

End-point event	ALL (*n* = 582)	TIL– group (*n* = 258)	TIL+ group (*n* = 324)	*p*
Total number of end-point events (n, %)	151 (25.9%)	40 (15.5%)	111 (34.3%)	<0.001
All-cause mortality events (n, %)	12 (2.1%)	5 (1.9%)	7 (2.2%)	0.85
Renal composite end-point events (n, %)	139 (23.8%)	35 (13.6%)	104 (32.1%)	<0.001

*Between the TIL– group and the TIL+ group.

**Figure 2.**
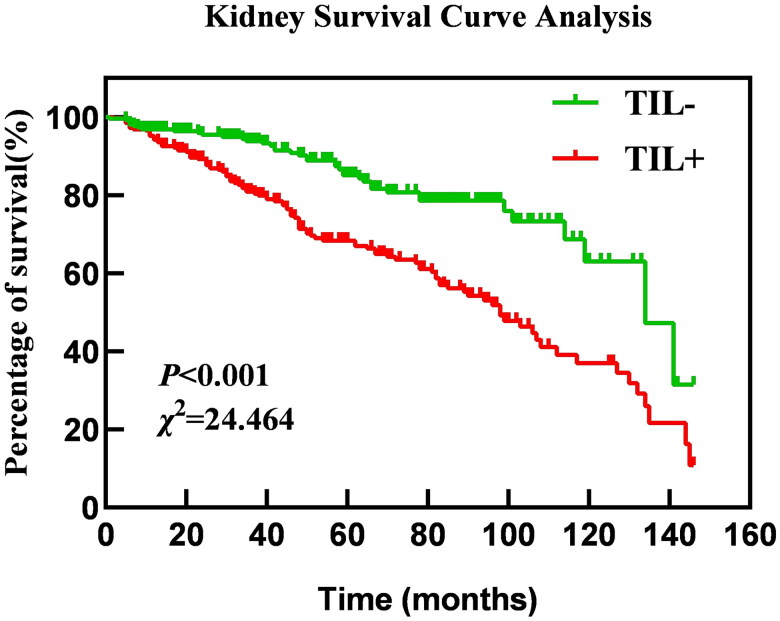
Impact of tubulointerstitial lesions on kidney outcomes in IMN patients.

Kaplan-Meier survival curve analysis, supplemented by log-rank tests, revealed significant differences in renal survival between idiopathic membranous nephropathy patients in the TIL+ and TIL– groups. Median renal survival was significantly shorter in the TIL+ group at 98 months compared to a median of 134 months in the TIL– group. Compared with IMN patients in the TIL– group, the TIL+ group had a lower cumulative renal survival rate, an earlier onset of poor renal prognosis, and a shorter renal survival time (*χ^2^* = 24.464, *p* < 0.01). The results are shown in Figure 2.

Cox regression model analysis showed that age, hypertension, triglycerides, high-density lipoprotein cholesterol, estimated glomerular filtration rate, and the presence of tubulointerstitial lesions were independently associated with renal outcomes. A detailed presentation of the Cox regression analysis is shown in Table 3.

**Table 3.** Cox regression analysis of prognostic factors in IMN patients.

Variable	Univariable Analysis		Multivariable Analysis	
	HR (95%CI)	*p*	HR (95%CI)	*p*
Female (versus male)	0.653 (0.465–0.918)	0.014	0.841 (0.561–1.261)	0.40
Age (per 1-year older)	1.016 (1.002–1.030)	0.029	1.018 (1.001–1.035)	0.03^a^
Hypertension (versus no)	2.395 (1.723–3.329)	0.000	2.140 (1.503–3.047)	<0.001^b^
Albumin (1 g/L increment)	0.972 (0.948–0.997)	0.027	0.992 (0.958–1.028)	0.67
Urinary Protein (1 g/24h increment)	1.170 (1.075–1.273)	0.000	1.035 (0.931–1.152)	0.52
Triglycerides (1 mmol/L increment)	1.100 (1.037–1.167)	0.001	1.074 (1.006–1.146)	0.03^c^
Total Cholesterol (1 mmol/L increment)	1.075 (1.001–1.154)	0.048	1.000 (0.909–1.100)	1.00
High-density Lipoprotein cholesterol (1 mmol/L increment)	0.776 (0.613–0.982)	0.034	0.757 (0.597–0.961)	0.02^d^
Low-density Lipoprotein Cholesterol (1 mmol/L increment)	1.133 (1.027–1.249)	0.013	1.092 (0.968–1.233)	0.15
Uric Acid (1 μmol/L increment)	1.002 (1.001–1.004)	0.005	1.001 (0.999–1.003)	0.23
eGFR (1 mL/min per 1.73 m² increment)	1.005 (1.001–1.010)	0.025	1.014 (1.009–1.019)	<0.001^e^
Immunosuppressive therapy (versus no)	1.708 (1.159–2.517)	0.007	1.497 (0.906–2.471)	0.12
Intimal thickening of small renal arteries (versus no)	1.800 (1.269–2.553)	0.001	1.423 (0.991–2.044)	0.06
TIL (versus no)	2.421 (1.684–3.480)	0.000	2.136 (1.459–3.125)	<0.001^f^

HR, hazard ratio; 95% CI, 95% confidence interval; eGFR, estimated glomerular filtration rate; TIL, tubulointerstitial lesions. ^a^Per 1 year older of age; ^b^Between hypertension and no hypertension. ^c^Per 1 mmol/L increase of triglycerides; ^d^Per 1 mmol/L increase of HDL-C; ^e^Per 1 mL/min/1.73m^2^ increase of eGFR; ^f^Between TIL and no TIL.

A logistic regression model was used to delineate the risk factors correlated with the presence of tubulointerstitial lesions in patients with idiopathic membranous nephropathy. The analysis identified hypertension, the presence of glomerulosclerosis or segmental glomerulosclerosis, and intimal thickening of small renal arteries as independent risk factors for the development of TIL (OR > 1). The results are presented in Table 4.

**Table 4.** Logistic regression analysis of factors associated with the presence of tubulointerstitial lesions in IMN patients.

Variable	Univariable Analysis		Multivariable Analysis	
	OR (95%CI)	*p*	OR (95%CI)	*p*
Female (versus male)	0.700 (0.500–0.979)	0.037	0.856 (0.592–1.237)	0.41
Age (per 1-year older)	1.030 (1.016–1.044)	0.000	1.011 (0.993–1.028)	0.23
Hypertension (versus no)	2.129 (1.521–2.979)	0.000	1.592 (1.099–2.307)	0.01^a^
Albumin (1g/L increment)	0.961 (0.938–0.985)	0.001	0.971 (0.942–1.000)	0.05
Urinary Protein (1 g/24h increment)	1.165 (1.067–1.272)	0.001	1.068 (0.957–1.191)	0.24
eGFR (1 mL/min per 1.73 m^2^ increment)	0.989 (0.985–0.994)	0.000	0.995 (0.990–1.001)	0.12
Pathological stage (versus stage III plus IV)	2.483 (1.261–4.888)	0.009	1.943 (0.959–3.939)	0.07
Globular/segmental glomerulosclerosis (versus no)	1.777 (1.256–2.513)	0.001	1.554 (1.040–2.255)	0.02^b^
Intimal thickening of small renal arteries (versus no)	1.536 (1.105–2.135)	0.011	1.499 (1.040–2.161)	0.03^c^

OR, Odds ratio; 95% CI, 95% confidence interval; eGFR, estimated glomerular filtration rate. ^a^Between hypertension and no hypertension; ^b^Between globular/segmental glomerulosclerosis and no globular/segmental glomerulosclerosis; ^c^Between intimal thickening of small renal arteries and no intimal thickening of small renal arteries.

## Discussion

In 2000, Prof. Haiyan Wang summarized studies including nearly 10 years of animal experiments and clinicopathology of human glomerular diseases and proposed that TIL damage is the ultimate pathway in the pathological development of most glomerular diseases, independent of glomerular inflammation [13]. The Boston Prospective Cohort Study reported that interstitial inflammatory cell infiltration, tubular atrophy, and interstitial fibrosis were independent predictors of renal failure. It concluded that TIL is an important determinant of the progression of chronic kidney disease [14]. While renal pathological damage in IMN has been extensively studied, focusing primarily on glomerular changes, the interplay between TIL and clinicopathological features as well as prognosis has been less explored.

Our study contributes to this discourse by examining a cohort of 582 IMN patients, revealing a TIL prevalence of 55.7%, a figure consistent with the findings reported by Stangou et al. [15]. However, the Chinese multicenter study showed that TIL was not only relatively common, but also relatively high in 19,337 cases of MN, in which the proportion of renal tubulointerstitial inflammatory cell infiltration was as high as 75.7%, tubular atrophy was 50.8%, and renal interstitial fibrosis was 17.7% [16]. The pathogenesis of TIL in chronic kidney disease remains elusive, with proposed mechanisms suggesting that under the influence of local inflammation, changes in renal hemodynamics and metabolism activate fibroblasts and induce a phenotypic shift of renal tubular epithelial cells toward mesenchymal cells, culminating in tubular atrophy and interstitial fibrosis [10,17].

Our study found that IMN patients with TIL had higher rates of hypertension, elevated triglycerides, blood urea nitrogen, and 24-h urine protein, as well as increased incidence of glomerulosclerosis and intimal thickening of small renal arteries. At the same time, these patients had decreased albumin levels and estimated glomerular filtration rate, observations consistent with previous studies [9,18,19]. Through logistic regression analysis, we identified hypertension, segmental glomerulosclerosis, and intimal thickening of small renal arteries as significant independent risk factors for TIL development in IMN patients.

Some opinions pointed out that hypertension is prone to arteriolar hyalinosis and glomerulosclerosis, renal tubules account for 80% of the total volume of the kidney and are closely connected with the per interstitial vessels. Therefore, elevated blood pressure can easily affect tubular damage. Bazzi et al. showed that in patients with glomerulonephritis combined with hypertension, renal tubular damage was more frequent with inflammatory cell infiltration in the interstitium, tubular atrophy, and more severe interstitial fibrosis than in patients with glomerulonephritis with normal blood pressure [20].

Several perspectives suggest a positive correlation between the extent of glomerulosclerosis and the severity of tubular atrophy and interstitial fibrosis [21]. Dumoulin’s research highlights that inflammatory mediators associated with glomerulosclerosis can damage renal tubular epithelial cells, potentially leading to tubulointerstitial fibrosis. This process may further induce capillary stenosis and ischemia, thereby exacerbating glomerulosclerosis [22].

In addition to glomerulosclerosis, other factors have been implicated in the development of TIL. Dyslipidemia is thought to cause lipid toxicity, characterized by ectopic accumulation of lipids in the renal tubules, which may induce inflammation, oxidative stress, and cellular damage. This may recruit and activate fibroblasts, promoting a pro-fibrotic environment and potentially leading to tubular epithelial cell apoptosis and interstitial fibrosis [23]. In addition, proteinuria has been suggested to contribute to tubulointerstitial inflammation and fibrosis through direct toxic effects, complement activation, and the initiation of inflammatory cascades that may generate oxidative stress and enhance apoptosis [24].

In our study, patients with TIL had lower disease remission rates and inferior treatment responses after six months of treatment compared to patients without TIL, highlighting the challenge of achieving clinical remission in this group. This is consistent with the findings of Horvatic et al. that IMN patients with tubular atrophy and interstitial fibrosis have a worse renal prognosis regardless of the use of immunosuppressive therapy. This suggests that prognosis may be influenced by non-immune mechanisms inherent to tubulointerstitial injury [8].

Our findings further revealed TIL as an independent risk factor for poor renal prognosis in IMN patients with a median follow-up of 45 months. TIL was associated with a 2.122-fold increased risk of end-point events compared to patients without TIL. Zhang et al. reported in a two-year follow-up study of 139 adult IMN patients that the risk of end-point events in IMN patients increased to 6.724-fold when the area of tubulointerstitial injury exceeded 25%. This supports the notion that tubulointerstitial injury is an independent risk factor for progression of IMN to end-stage renal disease [25].

Our study shows that idiopathic membranous nephropathy patients with tubulointerstitial lesions have more severe clinical manifestations and pathological changes. TIL stands out as an independent risk factor for poor renal function prognosis in IMN patients. In particular, hypertension, segmental glomerulosclerosis, and intimal thickening of small renal arteriesare identified as causes of TIL in IMN patients. These findings provide valuable theoretical guidance for clinical practice. They emphasize the importance of closely monitoring IMN patients with TIL and the need to explore targeted therapeutic strategies for TIL to improve the prognosis and quality of care for IMN patients.

However, the study has limitations. As a single-center retrospective analysis, it is subject to inherent bias. The validity of our findings should be confirmed by further prospective studies. In addition, the discussion on the pathogenesis of TIL is still in its infancy. The nature of TIL in MN patients, whether primary or secondary to the disease, remains to be elucidated in future research.

## Supplementary Material

Supplementary material.docx

## Data Availability

The data extracted from included studies and used for analyses are available upon reasonable request from the authors.
