# Supplementary material for: Renal tubulointerstitial lesions: a prognostic marker in idiopathic membranous nephropathy
Source: Ren Fail. 2025 May 22;47(1):2501379. doi: 10.1080/0886022X.2025.2501379 (PMC12100956; doi:10.1080/0886022X.2025.2501379)
Supplement: Supplementary material.docx [file IRNF_A_2501379_SM1470.docx]

**Table S1.** Comparison of treatment outcomes of IMN patients between the two groups.

| **Variable** | **TIL- group**  **(n=258)** | **TIL+ group (n=324)** | ***P*** |
| --- | --- | --- | --- |
| RAASI (n, %) | 77(29.8%) | 68(21.0%) | 0.01 |
| Immunosuppressive therapy (n, %) | 172(66.7%) | 248(76.5%) | 0.01 |
| Glucocorticoids+cyclophosphamide (n, %) | 128(49.6%) | 192(59.3%) |  |
| Glucocorticoids+calcineurin inhibitor (n, %) | 20(7.8%) | 36(11.1%) |  |
| Glucocorticoids+others (n, %) | 24(9.3%) | 20(6.2%) |  |
| 0thers(n, %) | 9(3.5%) | 8(2.5%) | 0.47 |

RAASI, renin-angiotensin-aldosterone system inhibitors. *Between the TIL- group and the TIL+ group.

**Table S2.** Comparison of baseline pathologic data between two groups of IMN patients.

| **Histopathological Feature** | **TIL**- **group (n=258)** | **TIL+ group (n=324)** | ***P*** |
| --- | --- | --- | --- |
| Pathological stage(number, percentage) |  |  |  |
| Stage I | 152(58.9%) | 153(47.2%) | 0.01 |
| Stage II | 94(36.4%) | 136(42.0%) | <0.001 |
| Stage III + IV | 12(4.7%) | 35(10.8%) | 0.01 |
| Globular/Segmental Glomerulosclerosis(number, percentage) | 76(29.5%) | 138(42.6%) | 0.001 |
| Intimal Thickening of Small Renal Arteries (number, percentage) | 111(43.0%) | 174(53.7%) | 0.01 |

*Between the TIL- group and the TIL+ group.

**Table S3.** Comparison of disease remission rates between two groups of IMN patients after 6 months of treatment.

| **Observation index** | **CR(n=64)** | **PR(n=234)** | **CR+PR(n=298)** |
| --- | --- | --- | --- |
| TIL- group (n=190) | 38(20.0%) | 108(56.8%) | 146(76.8%) |
| TIL+ group (n=240) | 26(10.8%) | 126(52.5%) | 152(63.3%) |
| *P* | 0.02 | 0.37 | 0.003 |

CR, complete remission; PR, partial remission. *Between the TIL- group and the TIL+ group.
